# Successful treatment of severe calcium channel blocker poisoning, new experience with the guidance of invasive hemodynamic monitoring in a 17-year-old girl: a case report

**DOI:** 10.1186/s13256-024-04345-1

**Published:** 2024-02-03

**Authors:** Amir Saeed, Amir Naghshzan

**Affiliations:** 1 Scientific Association of Intensive Care and ICU of Iran, Tehran, Iran; 2 Cardiovascular and Neonatology Research Center, Namazi Hospital, Shiraz University of Medical Sciences, Shiraz, Iran

**Keywords:** Case report, Calcium channel blocker poisoning, Pulse contour cardiac output

## Abstract

**Background:**

Calcium channel blocker poisoning is one of the most lethal cardiac drugs overdoses. Calcium and high-dose insulin infusion are the first-line therapy for symptomatic patients, and Intralipid emulsion infusion is useful for refractory cases.

**Case presentation:**

In this report, we describe a 17-year-old Iranian girl who took 250 mg of the drug for a suicidal attempt and presented with refractory hypotension and non-cardiogenic pulmonary edema treated successfully with the guidance of invasive hemodynamic parameters.

**Conclusion:**

For complicated cases, in addition to supportive care and adjuvant therapy such as high-dose insulin and Intralipid, it is mandatory to utilize advanced hemodynamic monitoring to treat hypotension in severe calcium channel blocker poisoning to guide the treatment.

## Background

Serum ionized calcium has the main role in cardiovascular function. It acts via cardiac conduction, contraction, and preservation of vascular tone. Calcium channel blocker (CCB) overdose is rare but lethal in cardiovascular medication-related drug overdose [1, 2].

Conventional and unconventional interventions were used to treat an adolescent who ingested a life-threatening dose of amlodipine [2]. Still, these studies are more limited in children, mainly due to the lower prevalence of this poisoning at an early age; therefore, we inevitably use adult studies when dealing with children referred with amlodipine poisoning.

Different treatment methods proposed for intoxication with this drug were based on amlodipine’s pharmacological and clinical findings. These treatments include gastric decontamination, calcium, glucagon, intravenous lipid emulsion, high-dose insulin therapy, sodium bicarbonate, vasopressors, and methylene blue [3]. In some instances, studies have used unconventional but somewhat effective treatments, including electrical cardiac pacing and venoarterial extracorporeal membrane oxygenation (VA-ECMO) [4]. However, on the basis of the patient’s condition, age, and the medical center facilities, a combination of these methods should be used. Due to the different treatment methods, the most critical part of deciding whether to continue treatment or use other treatment methods is to follow the patient’s clinical response, as well as to evaluate the hemodynamic findings during the treatment period.

Pulse contour cardiac output (PiCCO) is a less invasive, calibrated, continuous hemodynamic monitoring. This device uses transpulmonary thermodilution and the pulse contour analysis technique for volumetric measurements for preload, cardiac output, and stroke volume. For PiCCO monitoring, a central venous catheter should be inserted into the internal jugular or subclavian vein; then, a thermodilution arterial catheter is inserted into the femoral, radial, or brachial artery.

In PiCCO, the data relating to preload are global end-diastolic volume (GEDV) and intrathoracic blood volume (ITBV). Global ejection fraction (GEF) and cardiac output are two values for contractility measurements, and systemic vascular resistance (SVRI) indicates afterload. Lung parameters that indicate fluid overload are the extravascular lung water index (EVLWI) and pulmonary vascular permeability index (PVPI) [5].

Herein we describe a 17-year-old female patient with amlodipine poisoning with a refractory shock treated successfully with Intralipid in addition to calcium and a high dose of insulin; invasive hemodynamic monitoring parameters also guided inotropes and hydration.

## Case presentation

A previously healthy 17-year-old Iranian girl was taken to the emergency room (ER) with nausea, vomiting, vertigo, and a history of taking 250 mg amlodipine and 22.5 mg propranolol with suicidal attempt 4 hours before admission. At arrival, her vital signs were as follows: blood pressure (BP): 65/25, heart rate (HR): 62 beats per minute, and Glasgow Coma Scale (GCS):13/15. A nasogastric tube was inserted, and gastric lavage was done. Dopamine was started and increased to 20 micro (mcg)/kg/minute), and then norepinephrine was started. On arrival at the pediatric intensive care unit (PICU), BP was 82/35, and HR was 87 beats per minute, norepinephrine was increased up to 0.3 micro/kg/minute, but she was still hypotensive. She was intubated, a central line inserted into the jugular vein, an arterial line inserted into the femoral artery, and connected to the PiCCO monitor. The first hemodynamic parameters are listed in Table 1. According to the data, vasopressin was started.

**Table 1:** Hemodynamic parameters of days 1, 3, and 6 of PICU care

	Day 1	Day 3	Day 6
Heart rate	80	115	105
CVP + (mmHg)	9	14	10
SCVO2 Ω	90%	74	78
SBP (mmHg)	92	124	131
DBP (mmHg)	40	74	79
MAP (mmHg)	97	91	101
CI (L/min/m^2^)	7.1	5.8	4.6
ITBI	1263	720	672
ELWI (cc/kg) (3–7)	5	13	9
GEDI (cc/m^2^) (680–800)	1010	580	620
SVRI (1700–2400)	570	1320	1820
PPV (%) (0–10)	4	5	3
SSV (%) (0–10)	6	3	2
PVPI (1–3)	1.6	3.6	2.2

*CI* cardiac index, *CVP* central venous pressure, *DBP* diastolic blood pressure, *ELWI* extravascular lung water index, *GEDI* global end-diastolic index, *ITBI* intrathoracic blood volume index, *MAP* mean arterial blood pressure, *PPV* pulse pressure variation, *PPVI* pulse pressure variation index, *SBP* systolic blood pressure, *SCvO*_*2*_ central venous oxygen saturation, *SSV* stroke volume variation, *SVRI* systemic vascular resistance index

**Fig. 1:**
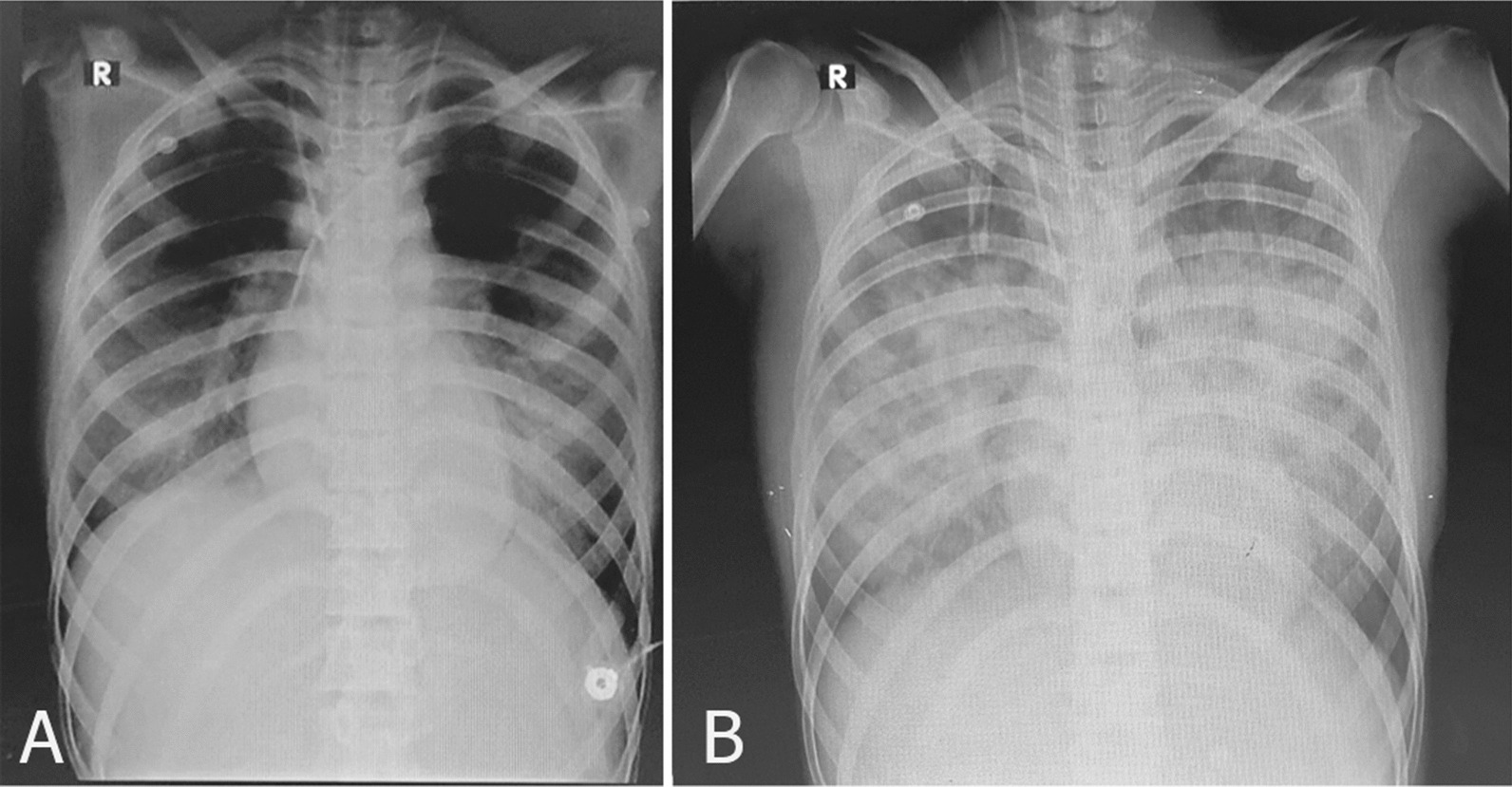
Changes of chest X-ray finding during PICU care, **A** day 1, **B** day 3. The progressive changes of chest X-ray and pulmonary edema during PICU care. These changes coincide with changes in the patient’s hemodynamics

To reach a euglycemic hyperinsulinemic state, the dextrose content of the maintenance intravenous fluid increased, and insulin infusion started from 0.5 IU/kg/hour and increased up to 6 IU/kg/hour (275 IU/hour), and so dextrose increased to 40% to keep glucose in the normal range. Calcium gluconate infusion was also started, and due to resistant hypotension, Intralipid 20%, 1.5 cc/kg was given as bolus in a minute, and infusion with the rate of 0.25 cc/kg/minute was also given for 2 hours, with this dose being repeated the following day due to refractory hypotension. During the PICU admission, she also presented with non-cardiogenic pulmonary edema (Fig. 1) and acute kidney injury (AKI) (creatinine level increased to 3.8). However, vasopressors were gradually tapered, and on day 9, the patient was extubated and tolerated, and 2 days later discharged from PICU without any sequela.

## Discussion and conclusion

This case report describes a young girl who intentionally took high doses of calcium channel blocker with hypotension and multiorgan failure and was successfully treated. In our patient, the first challenging issue was intubation and the risk of hypotension induced with a sedative drug. Ketamine is used safely for rapid sequence intubation in patients with hypotension due to positive sympathomimetic effects [6]. She was intubated with a low dose of ketamine and a high dose of rocuronium. Gastric decontamination and activated charcoal can be administered in the first 1–2 hours [7], but more than 4 hours had already passed, so this was not done.

There are two main classes of CCBs, and amlodipine is a type of long-acting dihydropyridine CCB that is mainly used for hypertension treatment. Cardiovascular instability resulting from calcium channel antagonist poisoning follows four parts: (1) negative inotropic effect (decreased contractility); (2) negative chronotropic effect (decreased heart rate); (3) decreased dromotropy presented as atrioventricular node blockade; and (4) decrease vascular tonicity that results in hypotension, and all might result in organ perfusion impairment; thus early detection and medical intervention are vital. The leading cause of hypotension in CCB toxicity is vasodilation, so one of the best diagnostic interventions is determining hemodynamic values and treating the patients according to those data.

The next step in our patient was stabilizing the cardiovascular system; high-dose dopamine infusion (20 mcg/kg/minute) and norepinephrine (0.3 mcg/km/minute) were not adequate for the management of the hypotension, and PiCCO helped us to decide what the next step to deal with the hypotension was (hydration versus adding a vasopressor or inotropes). Eventually, vasopressin was added and increased up to 0.6 units per minute. Then, high-dose insulin and dextrose in addition to Intralipid infusion were administered stepwise to decrease inotropes and vasopressin dose.

The first-line therapy for CCB toxicity is intravenous calcium and high-dose insulin (hyperinsulinemia euglycemia therapy) [7, 8, 14]. Increased intravascular calcium can increase the transmembrane flow of calcium, so cardiac contractility and vascular tone are increased. The high dose of insulin improves the inotropic effect; first 1 U/kg regular insulin is given as a bolus followed by infusion; its dosage can be titrated up to 10 U/kg/hour. With dextrose infusion to maintain euglycemia and achieve optimal hemodynamic effect [7], insulin infusion was increased up to 5 U/kg/hour to maintain acceptable blood pressure in our patient.

CCB intoxication has a negative chronotropic and dromotropic effect and may result in bradycardia and heart block. For patients who presented with unstable bradycardia or atrioventricular block, a pacemaker is a choice, but it was not needed in the case of our patient.

**Fig. 2:**
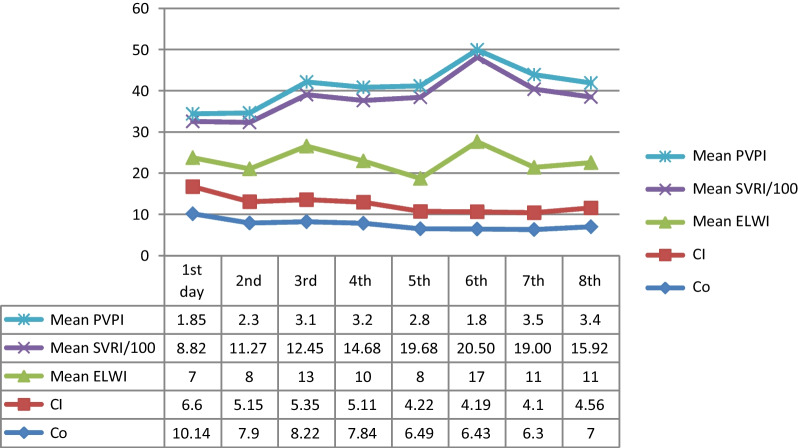
Mean value of hemodynamic parameters during PICU care (PICCO analysis), *CI* cardiac index, *CO* cardiac output, *ELWI* extravascular lung water index, *PPVI* pulse pressure variation index, *SVRI* systemic vascular resistance index

In patients with hypotension, inotrope selection is based on the type of shock and experts’ consensus recommendations, and using epinephrine and norepinephrine is recommended [7]. For cases with evidence of myocardial dysfunction, dobutamine could be added, but dopamine and vasopressin are not recommended. According to hemodynamic data (Table 1, Fig. 2), our patient had vasoplegic shock, so norepinephrine was started, then vasopressin was added, and their doses were effectively titrated. Although in some centers fluid hydration is recommended, our patient did not have any evidence of dehydration and volume responsiveness. In our patient, stroke volume variation (SVV) and pulse pressure variation (PPV) were less than 10% with normal cardiac index (CI, preserved cardiac function), and SVRI was low, so she was not volume responsive, and there was no need for hydration, thus vasopressin in addition to norepinephrine was added (Table 1, Fig. 2).

Intralipid has been used successfully to manage CCB poisoning as the second line for the refractory or in peri-arrest situations; there are some mechanisms of action for this drug such as lipid sink theory, positive inotropic effect, and improving fatty acid metabolism by cardiomyocytes [7, 9, 10]; the recommended dose is 1–1.5 cc/kg as a bolus; we used intralipid 20%, 1.5 cc/kg stat dose, and then 0.25 cc/kg/minute for 60 minutes. Then her BP increased and we could decrease the dose of inotropes. However, the next day her condition deteriorated, and the dose of vasopressors was increased, so we again administered Intravenous Lipid Emulsion (ILE) over 2 hours, and although it was about 26 hours after her admission, the hemodynamic parameters improved significantly (MAP and SVRI increased) (Table 1, Fig. 2); so there might be a role for delayed prescription of ILE in CCB overdose [10].

Amlodipine has a myocardial depressant effect via blocking L-type calcium channels to result in cardiogenic pulmonary edema. However, non-cardiogenic pulmonary edema also was seen in a few patients, but the mechanism of non-cardiogenic pulmonary edema is not precise. Some authors proposed that precapillary dilatation can increase pulmonary transudation and interstitial edema [11–13]. On day 3 of PICU admission, the pulmonary vascular permeability index and ELWI increased, and PaO2 decreased, so a chest X-ray was taken to evaluate pulmonary edema. Our patient had a normal echocardiogram, and PiCCO parameters were not indicative of volume overload or cardiac function impairment (CI and CO were normal), but PVPI and EVLWI were increased, which indicated increased permeability of pulmonary vessels and interstitial edema; all data were in favor of non-cardiogenic pulmonary edema.

Hemodynamic management, hydration, and selecting the type of inotrope or vasopressor are challenging in treating CCB poisoning. We cannot solely rely on clinical data that could be misguiding, so early transferring to ICU invasive hemodynamic monitoring in severe cases is vital.

In addition to supportive care and adjuvant therapy, such as high-dose insulin and Intralipid, it is mandatory to utilize advanced hemodynamic monitoring to treat hypotension in severe CCB poisoning to guide treatment.

## Data Availability

The datasets used and analyzed during the current study available from the corresponding author on reasonable request.

## Abbreviations

DBP: Diastolic blood pressure
ILE: Intravenous Lipid Emulsion
AKI: Acute kidney injury
BP: Blood pressure
CCB: Calcium channel blocker
CI: Cardiac index
CVP: Central venous pressure
ER: Emergency room
EVLWI: Extravascular lung water index
GCS: Glasgow Coma Scale
GEDI: Global end-diastolic index
GEDV: Global end-diastolic volume
GEF: Global ejection fraction
HR: Heart rate
ITBI: Intrathoracic blood volume index
ITBV: Intrathoracic blood volume
MAP: Mean arterial blood pressure
PiCCO: Pulse contour cardiac output
PICU: Pediatric intensive care unit
PPV: Pulse pressure variation
PPVI: Pulse pressure variation index
SBP: Systolic blood pressure
SCvO_2_: Central venous oxygen saturation
SSV: Stroke volume variation
SVRI: Systemic vascular resistance
SVV: Stroke volume variation
VA-ECMO: Venoarterial extracorporeal membrane oxygenation
